# The dental cavities of equine cheek teeth: three-dimensional reconstructions based on high resolution micro-computed tomography

**DOI:** 10.1186/1746-6148-8-173

**Published:** 2012-09-25

**Authors:** Susan Kopke, Nina Angrisani, Carsten Staszyk

**Affiliations:** 1 Institute of Anatomy, University of Veterinary Medicine Hannover, Foundation, Bischofsholer Damm 15, Hannover, D- 30173, Germany; 2 Small Animal Hospital, University of Veterinary Medicine Hannover, Foundation, Bünteweg 9, Hannover, D- 30559, Germany; 3 Institute for Veterinary Anatomy, -Histology and -Embryology, Faculty of Veterinary Medicine, Justus-Liebig-University Giessen, Frankfurter Str. 98, Giessen, D- 35392, Germany

**Keywords:** Horse, Equine dentistry, Dental anatomy, Dental roots, Pulp system, Root canal

## Abstract

**Background:**

Recent studies reported on the very complex morphology of the pulp system in equine cheek teeth. The continuous production of secondary dentine leads to distinct age-related changes of the endodontic cavity. Detailed anatomical knowledge of the dental cavities in all ages is required to explain the aetiopathology of typical equine endodontic diseases. Furthermore, data on mandibular and maxillary pulp systems is in high demand to provide a basis for the development of endodontic therapies. However, until now examination of the pulp cavity has been based on either sectioned teeth or clinical computed tomography. More precise results were expected by using micro-computed tomography with a resolution of about 0.1 mm and three-dimensional reconstructions based on previous greyscale analyses and histological verification. The aim of the present study was to describe the physiological configurations of the pulp system within a wide spectrum of tooth ages.

**Results:**

Maxillary teeth: All morphological constituents of the endodontic cavity were present in teeth between 4 and 16 years: Triadan 06s displayed six pulp horns and five root canals, Triadan 07-10s five pulp horns and four root canals and Triadan 11s seven pulp horns and four to six root canals. A common pulp chamber was most frequent in teeth ≤5 years, but was found even in a tooth of 9 years. A large variety of pulp configurations was observed within 2.5 and 16 years post eruption, but most commonly a separation into mesial and distal pulp compartments was seen. Maxillary cheek teeth showed up to four separate pulp compartments but the frequency of two, three and four pulp compartments was not related to tooth age (P > 0.05). In Triadan 06s, pulp horn 6 was always connected to pulp horns 1 and 3 and root canal I. In Triadan 11s, pulp horns 7 and 8 were present in variable constitutions. Mandibular teeth: A common pulp chamber was present in teeth up to 15 years, but most commonly seen in teeth ≤5 years. A segmented pulp system was found in 72% of the investigated teeth. Segmentation into separate mesial and distal pulp compartments was most commonly present. Pulp horn 4 coalesced either with the mesial pulp horns 1 and 3 or with the distal pulp horns 2 and 5.

**Conclusions:**

Details of the pulpar anatomy of equine cheek teeth are provided, supporting the continuous advancement in endodontic therapy. Numerous individual configurations of the pulp system were obtained in maxillary cheek teeth, but much less variability was seen in mandibular cheek teeth.

## Background

Despite recent studies describing the pulpar anatomy of equine cheek teeth [1,2], knowledge regarding physiological configurations and age-related changes of the pulp cavity remains incomplete [3]. Recently erupted mandibular and maxillary cheek teeth feature a single endodontic cavity comprising a very large common pulp chamber which connects all pulp horns [4]. Subsequent to the development of dental roots, root canals are formed and complete the morphological constituents of the pulp system. The continuous deposition of secondary dentine over all of the pulp cavity walls [5] leads to a segmentation of the pulp cavity into separate pulp compartments [6]. To our knowledge, no studies reporting on a lifelong progression of the pulpar segmentations in individual cheek teeth have been published.

Cheek teeth affected by pulpar or apical infection are still more commonly extracted than preserved [1,7,8]. Promising attempts were made to restore infected teeth by performing endodontic therapy [9-12]. However, the long-term success rates of apicoectomy followed by endodontic procedures range from 44% [13] to 86% [14]. One reason for poor success rates is insufficient knowledge of the variable pulpar morphology [3,11]. Indeed, in human dentistry detailed knowledge of the pulp horn and root canal configurations is an essential prerequisite for successful endodontic treatments [15] with impressive success rates of up to 95% being achieved [16-18].

Studies investigating pulpar anatomy have either been based on sectioned teeth, accepting inevitable loss of dental tissue [19-21] or clinical computed tomography, allowing sections as thin as 1 mm [1,2,4,22]. Even more precise results can be achieved by micro-computed tomography. This method provides a resolution of less than 0.1 mm, thus enabling the detection of even delicate interpulpar communications between pulp system components. The aim of the present study was to identify common physiological configurations of the pulp systems within different Triadan positions, to describe morphological features, and to analyse age-related changes.

## Methods

### Material

A total of 65 cheek teeth (30 maxillary teeth and 35 mandibular teeth) were extracted from the heads of 19 horses of different breeds obtained from several equine clinics in Northern Germany. Horses were subjected to euthanasia on human grounds for non-dental reasons. From each skull, varying numbers of cheek teeth were selected, ranging from one to eleven.

Maxillary and mandibular cheek teeth were analysed independently. The maxillary sample pool included seven Triadan 06s, eight Triadan 07s, one Triadan 08, six Triadan 09s, three Triadan 10s and five Triadan 11s. The mandibular sample pool comprised seven Triadan 06s, eleven Triadan 07s, two Triadan 08s, four Triadan 09s, three Triadan 10s and eight Triadan 11s. Due to their structural similarities [23] the central Triadan positions (Triadan 07–10) within the maxillary and the mandibular sample pool were grouped.

Horses` ages ranged from five to 24 years (median age 15 years). The age of 17 horses was determined by means of the equine ID card; the age of two horses was estimated using the ageing guides by Muylle (2005) [24] and Martin (2007) [25]. Owing to the staggered eruption times of cheek teeth, the dental ages were used for analyses and determined as published by Dixon (2005) [5]. The sample population included teeth between 1.5 and 23 years post eruption with a median age of 12 years in maxillary and 11 years in mandibular teeth. To calculate age-related changes of the pulp system, teeth were classified into one of the following four dental age groups:

Age group “young”: 0 – 5 years

Age group “middle-aged”: 5.5 – 12 years

Age group “old”: 12.5 – 17 years

Age group “senile”: >20 years

### Micro-computed tomography (μCT)

#### Preparation of cheek teeth

The maxillas and mandibles were disarticulated and divided along the midline, creating hemimaxillas and hemimandibles using a saber bone saw^a^. Cheek teeth were extracted intact along with surrounding tissues by sawing through the adjacent cheek teeth using a steel band saw^b^. To optimise imaging quality the samples were processed, reducing sample size and approaching original tooth size. These further adjustments were performed using a diamond-coated water-cooled band saw^c^. All teeth were fixed with 10% neutrally buffered formalin solution.

#### Scanning parameters

Each tooth was scanned individually using a XtremeCT^d^ with following scanning parameters: Cone beam, beam energy 60 kVp, electrical current 1 mA, resolution 82 μm (or 41 μm), integration time 439 ms (or 700 ms), algorithm optimised for bone, field of view 126 mm, maximal image matrix size 3072 x 3072 pixel. Scanning was performed in the coronal plane from the occlusal surface to the apex. Due to the isotropic voxel, slice thickness was equivalent to resolution, and thus 1000 to 2000 two-dimensional images were obtained.

#### Greyscale analyses and three-dimensional reconstruction

Two-dimensional images were evaluated using the operating software^e^. For means of thresholding, selected regions of pulp and adjacent dentine were transferred from a horizontal view to a histogram display. Pulp was delimited physically of dentine by bordering the pulp specific gauss distribution and in a following step the corresponding grey values for pulp were defined. By evaluating pulp in 202 images of six teeth the lower and upper mean values were equivalent with 0 to 1807 Hounsfield units. Reconstructions of three-dimensional models of the pulp system utilised greyscale thresholding based on previous greyscale analyses. Models of the pulp system were viewed using the operating software^e^ and evaluated visually for morphological features.

#### Validation of micro-computed reconstruction by means of ultra-structural microscopy

Two distinct regions of reference located at two pulp horns of one μCT model were identified to allow for consecutive measurements in μCT slices and in histological sections. Histological sample blocks were decalcified, sectioned and stained for examination (Masson-Goldner Trichrom). Both μCT slices and corresponding histological sections were evaluated on sagittal planes. The width of one μCT slice (82 μm) was covered by 13 histological sections, each measuring 6 μm. Measurements of pulp horn sizes were performed at three localisations, whereby four μCT models of different grey values (i.e. 1612 HU; 1807 HU; 2001 HU; 2148 HU) were compared with the corresponding pulp in histological sections. These evaluations revealed that a maximal value of 1800 HU for pulpar tissue served well. However, there was still a discrepancy of about 22 – 28%, with the μCT models being thicker than the histologically evaluated pulp horns. This discrepancy is due to using a predetermined value for reconstructions of the pulp system.

### Pulpar morphology

**Figure 1:**
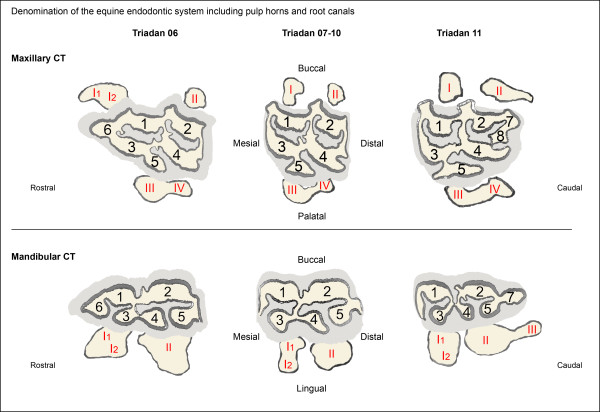
**Denomination of root canals and pulp horns for maxillary cheek teeth (top) and mandibular cheek teeth (bottom), adapted from Du Toit et al. (2008) [**[22]**].** The occlusal surface is illustrated by dentine (cream), cement (light grey) and enamel (dark grey). Root contours are schematically displayed adjacent to the occlusal surfaces. Positions of pulp horns are indicated with Arabic numerals (1 to 8). Positions of root canals are indicated with Roman numerals *(I to IV)* .

A recently modified equine endodontic numbering system was used for denomination of the pulp horns [22]. To address the root canals, a labelling was proposed using corresponding Roman numerals (for details see Figure 1). The morphology of the pulp systems was evaluated two- and three-dimensionally. To detect common age-related patterns of pulpar morphology, variations of the pulp systems were classified into categories and subsequently associations between categories and tooth age were calculated. To do so, models of the pulp cavity were evaluated regarding the segmentation into separate pulp compartments. A pulp compartment represents one functional unit of the pulp system in a cheek tooth, having no direct communication with other pulp compartments within the same tooth. One pulp compartment comprises at least one pulp horn and one root canal. All specimens were assigned to one of three degrees of pulpar segmentation:

**Common pulp chamber (CPC)** – all root canals and pulp horns communicate within the common pulp chamber

**Partial segmentation (PS)** – the pulp system is divided, but at least one pulp horn communicates with more than one root canal

**Maximal segmentation (MS)** – each pulp horn communicates with only one root canal

The degree of segmentation was then compared between young, middle-aged, old and senile cheek teeth. Associations between the status of pulpar segmentation (CPC, PS, MS) and age groups were calculated using Fisher Exact Probability Test for small sample sizes.

## Results

All structures of the tooth which were located occlusally of the bi- or trifurcation were considered as reserve crown, and all structures located apically to the bi- or trifurcation were considered as roots. Pulp horns were located within the reserve crown of teeth. The central cheek teeth (Triadan 07-10s) contained five pulp horns numbered 1 to 5. The second maxillary and mandibular premolars (Triadan 06s) contained six pulp horns with pulp horn number 6 additionally present at the mesial edge of teeth. The third molars (maxillary and mandibular, Triadan 11s) never displayed an additional pulp horn at the mesial side (referred to as pulp horn number 6). The third maxillary molars contained seven pulp horns with two additional pulp horns present at the distal edge, referred to as pulp horn numbers 7 and 8, whereas the third mandibular molars displayed six pulp horns with one additional pulp horn present at the distal edge, referred to as pulp horn number 7. Characteristically, pulp horns joined each other within the reserve crown showing different patterns of doing so and finally branched into root canals which terminated at their apical foramina. Some peculiar morphological features were seen, such as distinct double or triple connection canals between two pulp horns or elongated blind endings of former connections. In the present study all pulpar tissue located apically of the pulpar coalescences was defined as root canals, knowing that the coronal aspect of such root canals would still be located within the reserve crown. A varying number of root canals contributed to the coalesced pulp horns, with maxillary teeth showing a complex pattern of interpulpar communication compared to the rather simple pattern in mandibular teeth. In seven senile cheek teeth >20 years the pulp horns appeared completely filled with secondary dentine. Thus, rudimentary dental cavities comprising only root canals were observed. Therefore, all following descriptions of the pulp system refer to teeth up to 16 years in maxillary teeth and up to 17 years in mandibular teeth, respectively.

### Maxillary cheek teeth

#### Morphology of roots and root canals in maxillary cheek teeth

Three roots were seen in all teeth ≥2 years (25 of 26 teeth), with each of the two buccal roots containing one root canal. The elongated palatal root possessed two root canals in all teeth ≥4 years (20 of 26 teeth). These two palatal root canals (one mesial and one distal) contributed to separate pulp compartments in all but one tooth >5 years.

#### Segmentation into separate pulp compartments in maxillary cheek teeth

**Figure 2:**
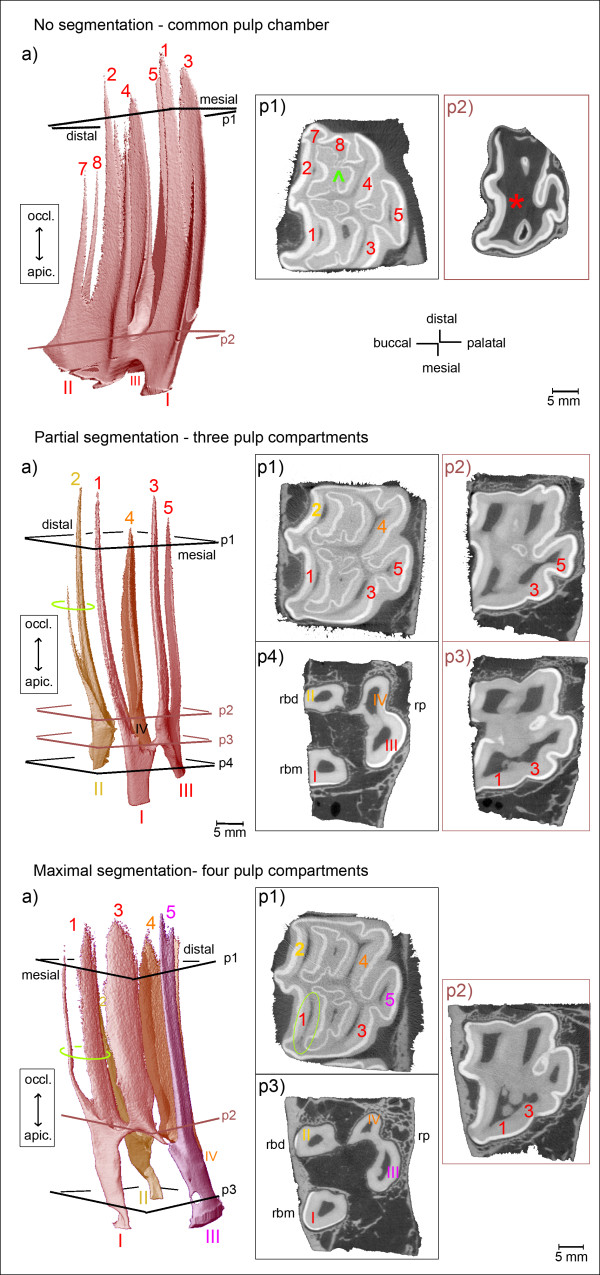
**Morphology of pulp horns and root canals in maxillary cheek teeth.** Arabic numerals (1 to 8): Pulp horns. Roman numerals (I to IV): Root canals. Colours indicate separate pulp compartments. **(a)** 3D models of the pulp cavity. Inserted planes p1 to p4 render the position of selected 2D μCT images, coloured planes indicate locations of connections. Sections **(p1-p4)** demonstrate cross-sectional shape and size of pulp horns and root canals. Dark grey: Pulp tissue; light grey: Dentine and cementum; white: Enamel; rbm = bucco-mesial root; rbd = bucco-distal root; rp = palatal root. **No segmentation** (Triadan 211, 2 years): A wide common pulp chamber (*) connects all pulp horns. Root canals are seen at early stage of development. **Partial segmentation** (Triadan 107, 5 years): Three separate pulp compartments are present. Root canals I and III contribute both to the branching pulp horns 1, 3 and 5. Root canal II solely contributes to pulp horn 2. Root canal IV solely contributes to pulp horn 4. Green ellipse: Pulp horn 2 is split up into a main and a delicate accessory branch. **Maximal segmentation** (Triadan 107, 8 years): Four solitary pulp compartments are present. Root canal I solely contributes to the coalesced pulp horns 1 and 3. Root canals II, III and IV solely contribute to pulp horns 2, 5 and 4, respectively. Green ellipse (a, p1): Pulp horn 1 is split up into a main distal and an accessory mesial branch.

**Figure 3:**
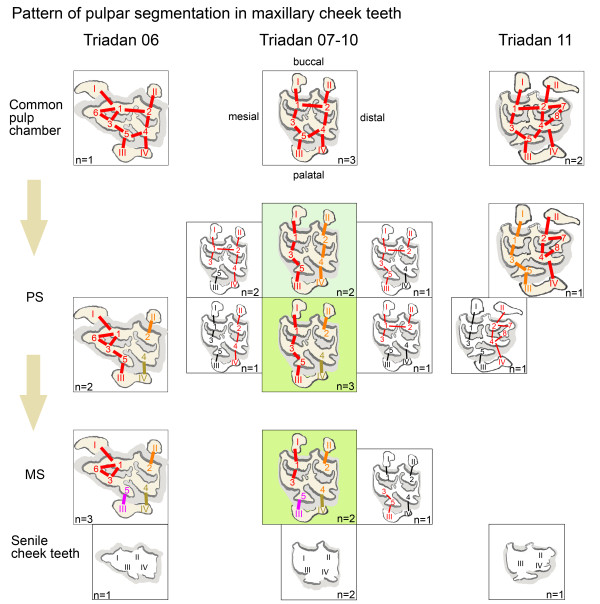
**Schematic illustrations of the numerous pulpar configurations with respect to commonly observed variations in maxillary cheek teeth of Triadan positions 06 to 11.** Degree of segmentation is displayed according to tooth age, starting with the non-segmented pulp cavity on top and the maximally segmented pulp cavity at the bottom. Configurations within each degree of segmentation are displayed in relation to their incidence. The most frequent configurations are highlighted by larger size and multi-colouring. Less common variations are smaller sized and simply coloured. The proposed pattern of pulpar segmentation with age is indicated in green. Connections are demonstrated by a straight line. Red is used for the largest pulp compartment within one tooth. Orange, ocher and purple differentiate between the second, third and fourth pulp compartment in a tooth.

The variation in pulp systems fell into one of three groups. The most common configuration within each group is illustrated and described in Figure 2. A common pulp chamber (CPC) which connects all root canals and pulp horns was found in six (23%) of 26 teeth. A partially segmented pulp chamber (PS) was present in 14 (54%) of 26 teeth. Hereby, the pulp cavity was divided into either two or three separate pulp compartments. The resultant pulp compartments did not always comprise the same communicating root canals and pulp horns. Instead, six variations of a PS were seen. The most common configuration of a PS was observed in five (36%) of 14 teeth: Root canal IV solely contributed to pulp horn 4, root canal II solely contributed to pulp horn 2, and root canals I and III contributed to the coalesced pulp horns 1, 3 and 5. In three (21%) of 14 teeth the distal root canals II and IV contributed to the distal pulp horns 2 and 4, and the mesial root canals I and III contributed to the mesial pulp horns 1, 3 and 5 (Figure 3). 

A maximally segmented pulp cavity (MS) was found in six (23%) of 26 teeth. According to the number of root canals in maxillary cheek teeth, four solitary pulp compartments were observed. Hereby, no more than two pulp horns were coalesced (Triadan 07-10s) and these being either pulp horns 1 and 3 or pulp horns 3 and 5. The most frequent MS was seen in five (83%) of six teeth, whereby the root canals II, III and IV solely contributed to the single pulp horns 2, 5 and 4, respectively, and root canal I solely contributed to the coalescing pulp horns 1 and 3. Pulp horn 3 was always coalesced with the adjacent pulp horn(s), even when solely derived from one root canal, and hereby most frequently with pulp horn 1 (five of six teeth) rather than pulp horn 5 (one of six teeth).

Regardless of the Triadan position, the separations of the pulp system were confined to the pulp horns 1 to 5. A major trend of segmentation into mesial and distal pulp compartments was seen in 16 (80%) of 20 teeth having a divided pulp system. Altogether, 12 different configurations of individual pulp compartments were found. Comparing the occurrence of individual pulp compartments it is worth mentioning that the following pulp compartments were most commonly observed: The solitary pulp compartment comprising pulp horn 4 and root canal IV (“4-IV”) was found in 13 (65%) of 20 teeth, “2-II” in 11 teeth (55%) and “5-III” in ten teeth (50%); followed by “I-1-3-5-III” in eight teeth (40%) and “I-1-3-III” in seven teeth (35%).

#### Pulp systems in maxillary Triadan 06s and Triadan 11s with respect to pulp horns 6, 7 and 8

**Figure 4:**
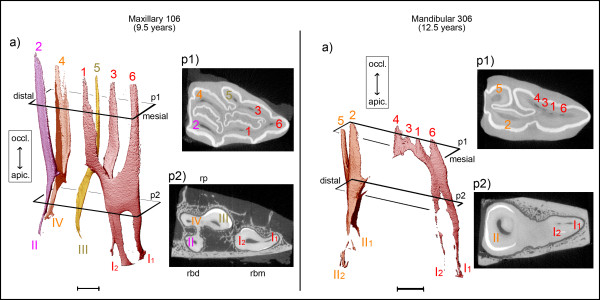
**Pulp horn 6 within the solitary mesial pulp compartment maxillary and mandibular second premolars (Triadan 06).** Scale bar: 5 mm. Maxillary 106, 9.5 years: Four pulp compartments are developed, with pulp horns 1, 3 and 6 derived from root canal I. Please note the furcation of root canal I into I1 and I2. Mandibular 306, 12.5 years: Two solitary pulp compartments are present. Pulp horn 6 is coalesced with distinct remnants of pulp horns 1, 3 and 4.

Compared to the central maxillary cheek teeth no further configurations of the pulp horns 1 to 5 were seen in the Triadan 06s and Triadan 11s. Instead, pulp horns 6, 7 and 8 were accessorily connected to the pulp compartments observed in the central Triadan positions. In all seven teeth of Triadan position 06 the mesial pulp horn 6 coalesced with its adjacent pulp horns 1 and 3. This coalescence formed just apical of the bottom of the mesial infundibulum. In the event of maximal pulpar segmentation, root canal I solely contributed to pulp horns 1, 3 and 6. Most commonly (in six of the seven teeth), root canal I released pulp horn 6 first, i.e. most apically, and then pulp horns 1 and 3 branched. Triadan 06s showed a furcation of the mesial root canal in three of four middle-aged teeth (Figure 4), with the furcation site being on the same level as the root formation and within the enamel layer (in two of the three teeth).

**Figure 5:**
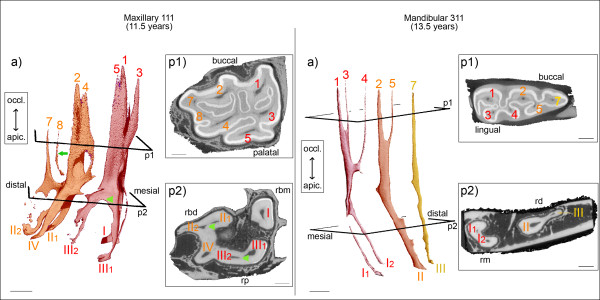
**Configurations of pulp horns and root canals in maxillary and mandibular third molars (Triadan 11).** Scale bar: 5 mm. Maxillary 111, 11.5 years: One mesial and one distal pulp compartment is present. Pulp horn 7 is connected to the adjacent pulp horn 2. Solid arrow (a): Isolated pulp horn 8. Arrowheads (a, p2): Furcation of root canals II and III. Mandibular 311, 13.5 years: Three pulp compartments are only developed in Triadan 11s. Root canal I solely contributes to pulp horns 1, 3 and 4; root canal II solely contributes to pulp horns 2 and 5. The third pulp compartment comprises root canal III which is connected to pulp horn 7. Both distal root canals II and III are placed in the elongated distal root.

The distally located pulp horns 7 and 8 in teeth of Triadan position 11 appeared smaller and shorter compared to pulp horns 1 to 5 (Figure 5). Pulp horn 7 was derived from root canal II and coalesced with pulp horn 2. Pulp horn 8 was derived from root canal IV and coalesced with pulp horn 4 (two teeth), was isolated from other pulpal tissue (one tooth, 11.5 years), or was absent (one tooth, 13.5 years). In one of two middle-aged teeth the bucco-distal root canal (II) as well as the palatal root canal (III) was forked, with the furcation being located slightly occlusally to the roots and the enamel layer (Figure 5).

#### Degree of pulpar segmentation and tooth age in maxillary cheek teeth

**Figure 6:**
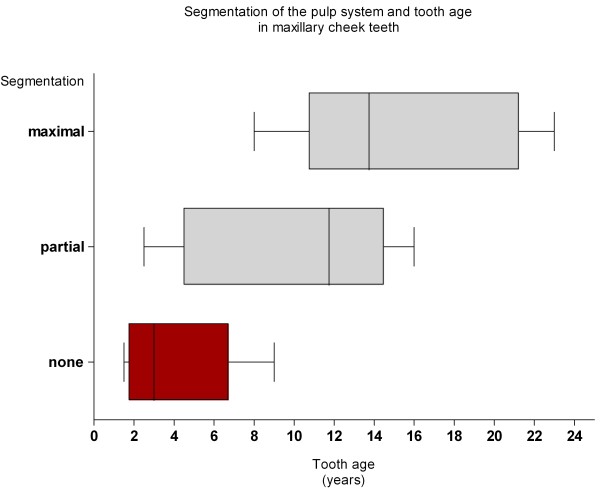
**Boxplots of the tooth ages of maxillary cheek teeth assigned to three degrees of segmentation.** The shaded box represent the interquartile range (25%, 75%), the vertical line the median and the whiskers the range.

Tooth ages and the observed number of pulp compartments are documented in Table 1. All teeth ≤2 years showed a common pulp chamber. The youngest tooth having two separate pulp compartments was 2.5 years, followed by a 3-year-old tooth having three separate pulp compartments, and finally four solitary pulp compartments were found in teeth ≥8 years (Figure 6). The occurrence of the common pulp chamber was age-related, with the risk ratio of a common pulp chamber being present in teeth ≤5 years compared to teeth >5 years being 9.44 (95% CI >1). The degree of segmentation was independent of age (P = 0.3913). Thus, the probability of teeth ≤16 years having two, three or four pulp compartments did not differ significantly between the three age groups. Within age group “young” (tooth age 1.5 – 5 years) the common pulp chamber was the most frequent configuration pattern and was seen in five (56%) of the nine teeth. In teeth >5 years the common pulp chamber was seen in one tooth aged 9 years, but all other teeth among the middle-aged and old cheek teeth (tooth age 8 – 16 years) had a segmented pulp cavity. 

**Table 1:** Observed pulp compartments in maxillary cheek teeth of Triadan position 06 to 11

		**Number of teeth**			
		**No segmentation (CPC)**	**Partial segmentation (PS)**		**Maximal segmentation (MS)**
**Tooth age (years)**	**Total no. of teeth in group**	**Common pulp chamber**	**Two pulp compartments**	**Three pulp compartments**	**Four pulp compartments (all solitary)**
1.5 – 2	2	2	0	0	0
2.5 – 4	5	2	1	2	0
4.5 – 5	2	1	0	1	0
8	1	0	0	0	1
9 – 9.5	3	1	0	1	1
11 – 12	4	0	1	2	1
12.5 – 14	5	0	1	2	2
14.5 – 16	4	0	3	0	1
>20	4	0	0	0	4

Intercommunication between all pulp horns was observed up to 9 years post eruption. Partially segmented pulp systems (PS) were seen in teeth ≥ 2.5 years post eruption. Configurations of PS varied – solitary, medium sized or large separate pulp compartments were combined. Four pulp compartments (MS) were found in teeth ≥8 years post eruption. In all teeth >20 years rudimentary dental cavities were observed.

### Mandibular cheek teeth

#### Morphology of roots and root canals in mandibular cheek teeth

Formation of two roots was seen in all teeth >2 years (28 of 32 teeth). One root canal within each root was found in all teeth between 2.5 and 4 years. Two branches of the mesial root canal were seen in one tooth aged 4.5 years, but in all teeth ≥7 years (22 of 32 teeth). Within the distal root the undivided root canal was found in most teeth <10 years (11 of 12 teeth), whereas in teeth ≥10 years branching of the distal root canal was most commonly present (12 of 16 teeth). Comparing the branching sites of the root canals within mesial and distal roots, division of the distal root canal was located further apically and in most cases was seen even apically of the enamel extension, with this being independently of tooth age. In Triadan 11s an additional root canal within the elongated distal root was seen in teeth >8 years.

#### Segmentation into separate pulp compartments in mandibular cheek teeth

**Figure 7:**
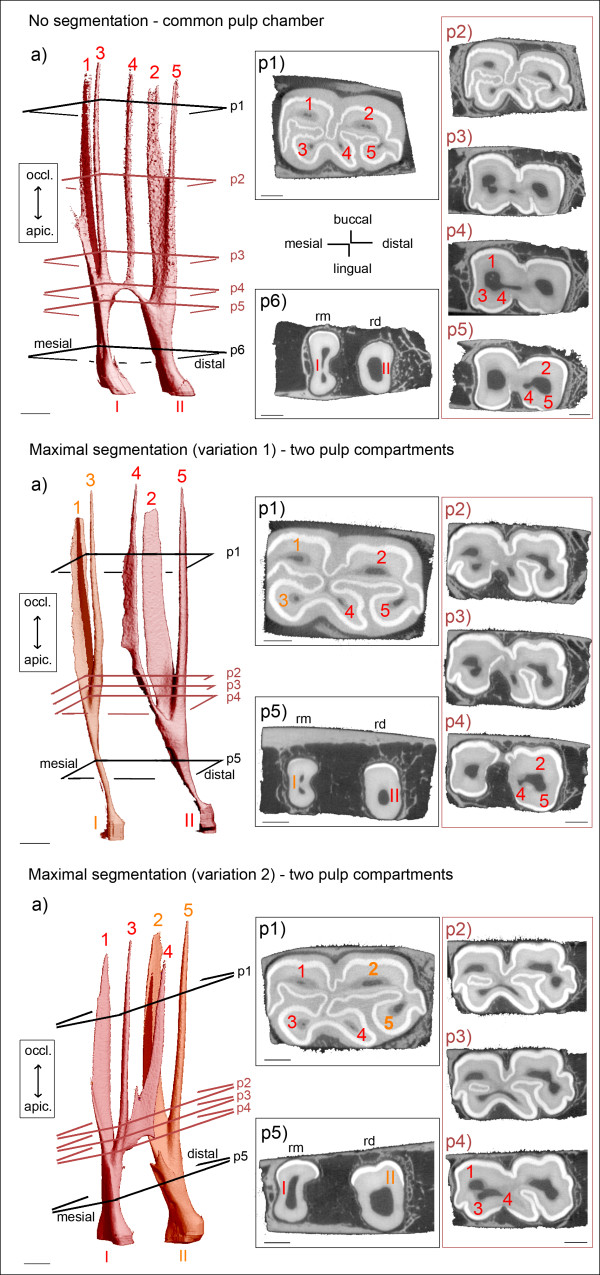
**Morphology of pulp horns and root canals in mandibular cheek teeth.** Arabic numerals (1 to 7): Pulp horns. Roman numerals (I to III): Root canals. Colours indicate separate pulp compartments. **(a)** 3D images of the pulp cavity. Inserted planes p1 to p6 render the position of selected 2D μCT images, coloured planes indicate locations of connections. Sections **(p1 - p6)** demonstrate cross-sectional shape and size of pulp horns and root canals. Dark grey: Pulp tissue; light grey: Dentine and cementum; white: Enamel; rm = mesial root, rd = distal root. Scale bar: 5 mm. **No segmentation** (Triadan 409, 15 years): A narrow common pulp chamber connects all five pulp horns. Two distinct root canals are developed. **Maximal segmentation (1)** (Triadan 407, 7 years): Two pulp compartments are present, with pulp horn 4 included in the distal pulp compartment. Root canal I solely contributes to pulp horns 1 and 3. Root canal II solely contributes to pulp horns 2, 4 and 5. **Maximal segmentation (2)** (Triadan 407, 5 years): Two pulp compartments are displayed, with pulp horn 4 connected to the mesial pulp compartment. Root canal I solely contributes to pulp horns 1, 3 and 4. Root canal II solely contributes to pulp horns 2 and 5.

**Figure 8:**
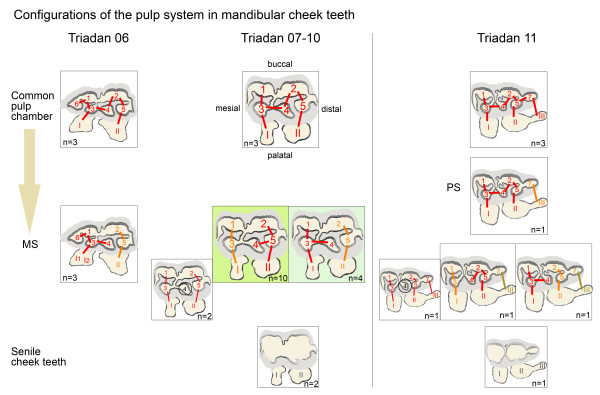
**Schematic illustrations of pulpar configurations with respect to commonly observed variations in mandibular cheek teeth of Triadan positions 06 to 11.** The most frequent configurations are highlighted in green. Less common variations are smaller sized. Connections are demonstrated by a straight line. Red is used for the largest pulp compartment. Orange represents the second and ocher the third pulp compartment within one tooth. Encircled numerals represent either isolated pulp horns or pulp horns which are completely filled with secondary dentine. In Triadan 06s, pulp horn 6 was always connected to the mesial pulp compartment. In Triadan 11s, pulp horn 7 was always connected to the additional root canal III.

**Figure 9:**
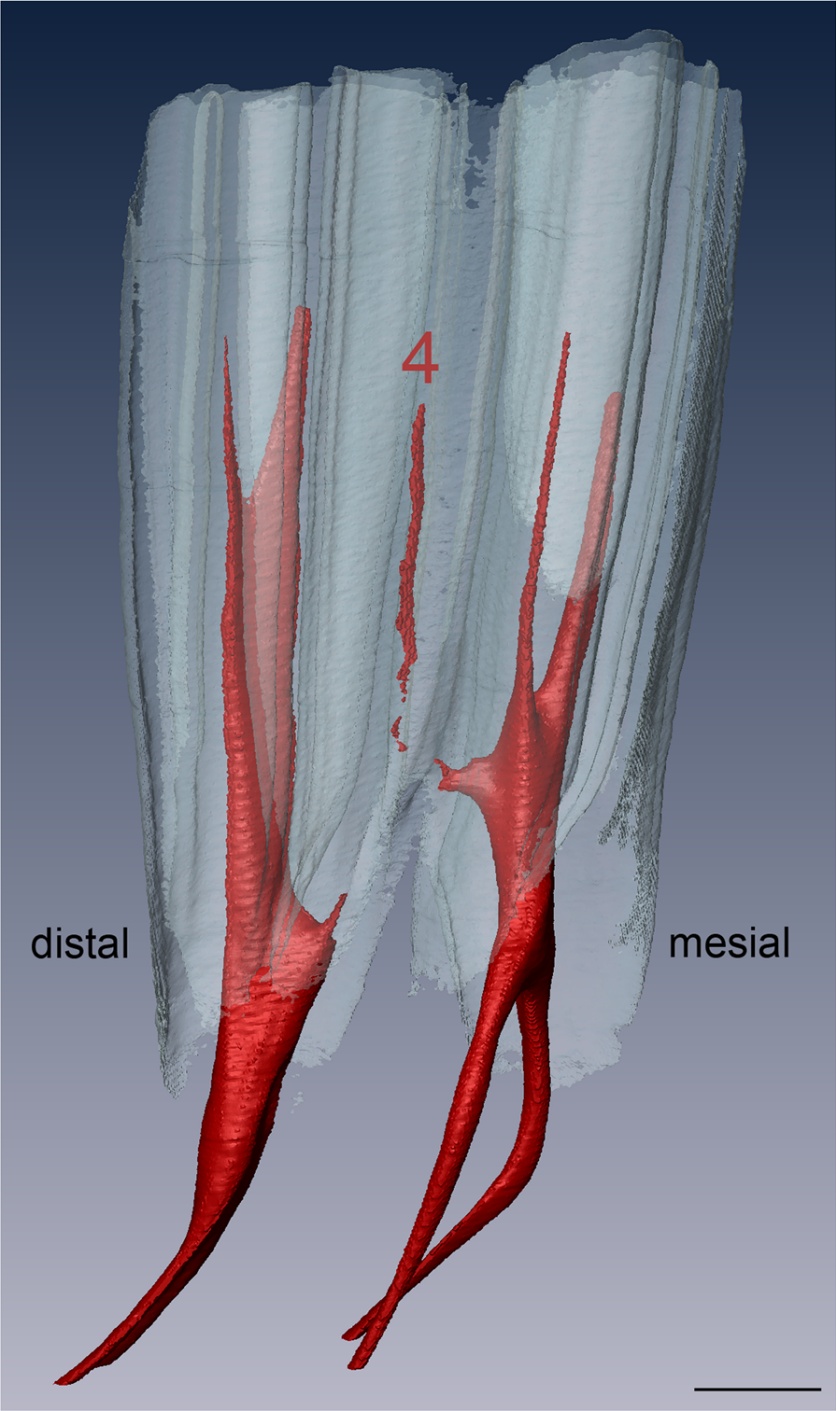
**Isolated pulpar tissue.**^**f**^ Two pulp compartments are developed, with pulp horn 4 being isolated (Triadan 309, 16 years). Scale bar: 5 mm.

A common pulp chamber (CPC) connecting all pulp horns and root canals was observed in nine (28%) of 32 teeth. The maximally segmented pulp chamber (MS), i.e. each pulp horn is derived by only one root canal, was found in 22 (69%) of the 32 teeth. The solitary pulp compartments of mandibular teeth comprised up to three coalesced pulp horns being derived from one root canal (Figure 7). The most common configuration of a maximally segmented pulp system was seen in 11 (50%) of 22 teeth, with the mesial root canal I solely contributing to the mesial pulp horns 1 and 3, and the distal root canal II solely contributing to pulp horns 2, 4 and 5. The most common variation was found in eight (36%) of 22 teeth, whereby pulp horn 4 was coalesced with the mesial pulp horns (Figure 8). The least frequent variation of a maximally segmented pulp system was observed in three (14%) of 22 teeth, with pulp horn 4 being isolated from any pulp compartment or being absent in spite of definite encompassing pulp cavity formations (Figure 9). All three variations indicated that pulp horn 4 was the only discontinuity within a segmented pulp cavity. 

#### Pulp systems in mandibular Triadan 06s and Triadan 11s with respect to pulp horns 6 and 7

Compared to the central mandibular cheek teeth no further configurations of the pulp horns 1 to 5 were seen in the second premolars and the third molars. In all six teeth of Triadan position 06, the mesial pulp horn 6 was accessorily connected to the adjacent pulp horns 1 and 3, with the connection site being located apically of the coalescence of pulp horn 1 and 3. In all Triadan 06s having a segmented pulp cavity, root canal I solely contributed to the coalesced pulp horns 1, 3, 4 and 6 (Figure 4). In three of seven teeth of Triadan position 11 the distally located pulp horn 7 was connected to the common pulp chamber. However, in all four specimens having roots developed an additional distal root canal III was seen within the distal root. Thus, a third pulp compartment was observed. This typical third pulp compartment was separate in all four teeth and was composed of pulp horn 7 being solely derived from root canal III (Figure 5).

#### Degree of pulpar segmentation and tooth age in mandibular cheek teeth

**Figure 10:**
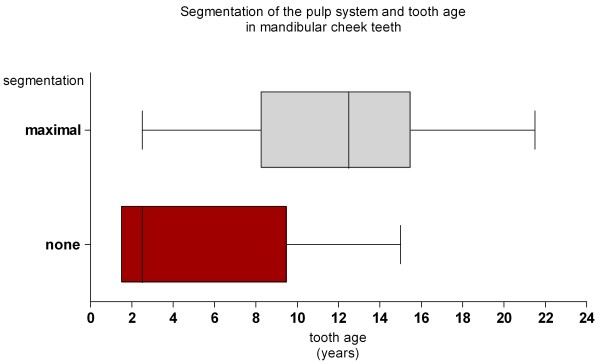
**Box plots of the tooth ages of mandibular cheek teeth assigned to none and maximal segmentation.** The shaded box represents the interquartile range (25%, 75%), the vertical line the median and the whiskers the range.

Tooth ages and the observed number of pulp compartments are documented in Table 2. All teeth ≤2 years showed a common pulp chamber. The youngest tooth having two separate pulp compartments was 2.5 years (Figure 10). Within age group “young” (tooth age 1.5 – 5 years) the common pulp chamber was the most frequent configuration pattern and was seen in six (60%) of ten teeth. In teeth >5 years (n = 22) the common pulp chamber was seen in two teeth aged 9.5 years and in one tooth aged 15 years. The occurrence of the common pulp chamber was age-related (P = 0.0125), with the relative risk of a common pulp chamber being present in teeth ≤5 years compared to teeth >5 years being 4.4 (95% CI >1). In middle-aged and old cheek teeth (tooth age 7 – 17 years) a maximally segmented pulp cavity was most commonly observed and seen in 18 (82%) of 22 teeth. The probability of cheek teeth having a maximally segmented pulp cavity increased with age (P = 0.0463). 

**Table 2:** Observed pulpar segmentation in mandibular cheek teeth of Triadan position 06 to 11

		**Number of teeth**	
		**No segmentation (CPC)**	**Maximal segmentation (MS)**
**Tooth age (years)**	**Total no. of teeth in group**	**Common pulp chamber**	**Two and three pulp compartments (all solitary)**
1.5 – 2	4	4	0
2.5 – 4	4	2	2
4.5 – 5	2	0	2
7 – 8	2	0	2
8.5 – 10	5	2	2
11 – 12	4	0	4
12.5 – 14	5	0	5
14.5 – 17	6	1	5
>20	3	0	3

In all teeth ≤2 years a common pulp chamber was seen. In all teeth >20 years rudimentary dental cavities were present.

## Discussion

The pulp cavity of equine maxillary and mandibular cheek teeth is subject to profound age-related changes throughout its life span [5]. Due to continuous deposition of secondary dentine around the walls of the dental cavity, and distinct growth processes within the apical third of the tooth, the pulp chamber narrows and subsequently divides into separate pulp compartments. In the present study, variable configurations of the pulp cavity were observed even in teeth of the same age and the same Triadan position. However, major trends of pulpar segmentation were identified for mandibular and maxillary cheek teeth. Generally, the number of separate pulp compartments within one tooth increased significantly with age. Interestingly, there was a trend of premolars having less interpulpar communication in young teeth and more often solitary pulp compartments in aged teeth compared to molars. Isolated and completely filled pulp horns were found only in the third molars (pulp horns 4 and 8 in mandibular and maxillary teeth, respectively) and in mandibular Triadan 09s (pulp horn 4). Senile teeth >20 years showed rudimentary endodontic cavities due to advanced abrasion and attrition, with interpulpar communications being absent.

### Common pulp chamber

The common pulp chamber was found in six (20%) of 30 maxillary teeth and in nine (26%) of 35 mandibular teeth. However, a similar study found a higher prevalence of 26% in maxillary teeth, but a lower prevalence of 14% in mandibular teeth [2]. Presumably, small intermissions between pulp compartments in maxillary teeth might not be detected by clinical computed tomography having a resolution of 1 mm, which was used by Windley et al. (2009) [2]. Nonetheless, Windley et al. (2009) [2] stated the common pulp chamber to be the most frequent pulpar configuration in maxillary cheek teeth which is in line with data of our study.

It has been shown that pulp horn coalescences are more common in younger horses [1,4]. The mean dental age of maxillary teeth displaying the common pulp chamber was 4 years in the present study. Dacre et al. (2008) [1] observed pulpar communication in teeth having a mean dental age of only 2 years. Furthermore, the oldest maxillary tooth in the present study showing the common pulp chamber was 9 years, whereas Windley et al. (2009) [2] observed the common pulp chamber only up to 6 years post eruption. The mean dental age of mandibular teeth displaying the common pulp chamber was 5 years in the present study, this being similar to results documented by Dacre et al. (2008) [1], who found the mean dental age of teeth having pulpar communication to be 4.5 years. The common pulp chamber of mandibular teeth was seen up to 15 years post eruption in the present study. Our results differ greatly from studies of Kirkland et al. (1996) [4] and Windley et al. (2009) [2], who found the common pulp chamber only in teeth <6 years, and <2 years, respectively. These differing results are probably one effect of the μCT inherent higher resolution compared to clinical computed tomography (used by Kirkland et al. (1996) [4] and Windley et al. (2009) [2]), which enabled us to detect even delicate and curved endodontic cavities.

### Segmented pulp cavity

Once the common pulp chamber was divided into separate pulp compartments, configurations of the dental cavities varied and appeared unpredictable [2]. Results indicate that in mandibular teeth >5 years solitary pulp compartments are frequently present, with still a minor risk left of teeth having all pulp horns connected in middle-aged and even in old teeth. Apparently, segmentation of the maxillary pulp system into separate solitary pulp compartments increased gradually between 2.5 and 8 years post eruption. As shown, maximal segmentation of the pulp cavity was not observed even in some aged teeth. It probably would be of clinical interest to determine the factors causing delayed pulpar segmentation in cheek teeth. Despite the observed gradual beginning of pulpar segmentation, further separation of the pulp system, perhaps once the root canals have been developed, may proceed slowly. However, to fully consider this question, further studies are needed providing closely spaced age groups between 5 and 20 years of tooth age, including large numbers of specimens.

In maxillary teeth ≤16 years having a segmented pulp cavity eight different configurations were observed in the present study. Hereby, two, three or four separate pulp compartments were displayed, with pulp horns appearing variably coalesced. However, in accordance with findings of Windley et al. (2009) [2], two configurations were most commonly observed (in ten (50%) of 20 teeth with segmented pulp systems): a) the pulp horns 1-3-5, 2 and 4 were separately derived from root canals I-III, II and IV, respectively, and b) pulp horns 1–3, 5, 2 and 4 were solely derived from root canals I, III, II and IV, respectively. The second most common configuration was observed in three teeth (15%), whereby “I-1-3-5-III” and “II-2-4-IV” was seen. In general, all teeth had at least two coalesced pulp horns present, these being most commonly the mesial pulp horns 1 – 3 (in 19 of 20 divided pulp systems; 95%) and the mesial pulp horns 3 – 5 (in 10 of 20 divided pulp systems; 50%). Similarly, observations of sectioned teeth by Dacre et al. (2008) [1] and studies of donkey teeth by Du Toit et al. (2008) [22] revealed coalescence of pulp horns 3 – 5 to be mostly present. It is noteworthy that separation of the pulp cavity into mesially and distally located pulp compartments occurred in 16 (80%) of 20 maxillary teeth. Conversely, coalescence of a mesial and a distal pulp horn was only seen in four (20%) of 20 divided pulp systems, this always being the buccal pulp horns 1 – 2. The maxillary pulp horn 3 was missing a corresponding root canal and therefore was always connected to pulp horns 1 and/or 5. Windley et al. (2009) [2] aptly mentioned that pulp horn 3 was split and appeared to be derived from the mesiobuccal and palatal roots.

Within segmented mandibular pulp systems four different configurations were observed. As already documented by Kirkland et al. (1996) [4], only two separate pulp compartments were developed in teeth of Triadan positions 06 to 10. In teeth of Triadan position 11, configurations of the pulp system within pulp horns 1 to 5 did not display additional patterns of segmentation, but a third solitary pulp compartment was established distally, with pulp horn 7 being derived from root canal III and terminating at an additional apical foramen. Conversely, Windley et al. (2009) [2] observed pulp horn 7 as being always connected to pulp horns 4 and 5 with no additional distal root canal being present.

Generally, in 32 (100%) mandibular teeth ≤17 years, distinct pulp horns were always connected: The mesial pulp horns 1 and 3 were coalesced as well as the distal pulp horns 2 and 5. However, Du Toit et al. (2008) [22] found only 37.5% of teeth having both these communications present, whereas Dacre et al. (2008) [1] only saw this pattern in one tooth. In the present study both coalescences were part of solitary pulp compartments. Furthermore, pulp horn 4 (if present and not isolated) was either connected to the distal or to the mesial pulp compartment, more commonly being part of the distal pulp compartment (11 of 19 teeth; 58%) compared to the mesial pulp compartment (eight of 19 teeth; 42%). Windley et al. (2009) [2] even documented the distal coalescence of pulp horn 4 in 68% of mandibular cheek teeth. Interestingly, only pulp horn 4 was seen to be isolated or absent, as observed by Windley et al. (2009) [2], this being found in three teeth >8 years. In accordance with other studies no further interpulpar communication between the mesial and the distal pulp compartment was seen [1,2,4]. This finding can be explained by the pronounced buccal enamel infolding (ectoflexid) in mandibular teeth reaching further apically compared to the lingual infolding (linguaflexid). Consequently, apart from pulp horn 4, the gross anatomy of mandibular teeth does not permit any further connection between the mesial and distal pulp compartments. If pulp horn 4 in mandibular teeth is exposed, either the mesial or the distal pulp cavity might be affected, or this pulp tissue could be necrotic due to isolation of the pulp horn. In turn, if exposure of pulp horns other than number 4 is diagnosed, their corresponding root canal can be clearly determined.

### Apical morphology

Root canals can take various pathways to the apex including branches, divisions and rejoining, as has been described in great detail in human dentistry. Features describing variations of the root canals comprise accessory, lateral and furcation canals, canal orifices, apical deltas and apical foramina [15]. Many features of the root canals were recently observed in mandibular teeth [21]. In the present study the mesial and distal root canal of mandibular teeth dispersed into two branches of varying length, width and route with gradually increasing age. Windley et al. (2009) [2] found two branches of the distal root canal even in all teeth >10 years and accordingly observed the root canals to unite occlusally to the roots. Kirkland et al. (1996) [4], who observed teeth up to eight years, found two root canals in the mesial root and one root canal in the distal root in teeth >5 years. Westenberger (2002) [20] observed a varying total number of up to three root canals in mandibular cheek teeth. In general, root canals of maxillary teeth appeared shorter compared to mandibular root canals, and were not divided except for the second premolars and the third molars. These teeth are macroscopically of larger size, thus giving space for additional pulpar tissue within roots in order to maintain the nutritional function.

### Clinical aspects

Occlusal pulpar exposure is associated with previous pulpar insults followed by reduced or ceased deposition of dentine [26-28]. Multiple pulpar exposure (two and more pulp horns) is considered to indicate that the entire endodontic system is affected [29]. However, Joest (1970) [30] stated that due to the pulp configuration a partial pulpitis does not necessarily affect all pulp horns. Casey and Tremaine (2010) [31] showed multiple defective secondary dentinal areas to be more prevalent in diseased mandibular teeth compared to maxillary teeth. Assumingly, this might be due to developing solitary pulp compartments which are composed of only one pulp horn and one root canal more frequently in maxillary teeth, compared to mandibular teeth which display solitary pulp compartments comprising up to three coalesced pulp horns. Furthermore, the most commonly defective area identified by Casey and Tremaine (2010) [31] was pulp horn 2 in maxillary teeth. This result tallies with findings in the present study whereby the solitary pulp compartment comprising only pulp horn 2 and root canal II was found in 11 (55%) of 20 teeth having a divided pulp cavity, thus being the second most common individual pulp compartment observed in maxillary cheek teeth.

As reported in clinical studies the outcome of endodontic therapy was more successful in mandibular compared to maxillary teeth [11,13,32]. A study by Carmalt and Barber (2004) [33] even reported only one root being affected in 100% of 14 mandibular teeth having a mean age of 5.3 years. Similar to this finding, in 69% of 32 mandibular teeth of the present study, which had a mean age of 9 years, the pulp cavity was maximally divided, presumably preventing the spread of pulpar infection to adjacent pulp compartments via mesio-distal connections. However, only in 23% of 26 maxillary teeth of the present study (mean age: 9 years) was maximal segmentation present, thus pulpar infection could spread to unaffected pulps via variable connections in the majority of teeth. Despite such high degree of variable pulpar configurations in maxillary teeth, we observed a division of the pulp cavity into mesially and distally located pulp compartments similar to mandibular teeth, with the solitary pulp compartments “4-IV”, “2-II” and “5-III” being most commonly present. Presumably, the knowledge [11] and selected treatment of solitary pulp compartments could simplify endodontic procedures and therefore improve clinical outcomes.

## Conclusions

The present study has highlighted variable pulpar configurations of equine cheek teeth and documented profound age-related changes to the pulp system. A wide range of tooth ages displaying individual configurations was seen, and major trends were observed. It might be of great value to the clinician to be acquainted with the general configurations of the pulp system, since knowledge optimises prognostic evaluations of diseased teeth.

## Endnotes

^a^saber bone saw, type EFA 61, Schmid & Wezel GmbH & Co. KG, Maulbronn, Germany; ^b^steel band saw, type K 420, Kolbe GmbH, Elchingen, Germany; ^c^diamond-coated band saw, type MBS 220/E, Proxxon GmbH, Föhren, Germany; ^d^XtremeCT, Scanco Medical AG, Brüttisellen, Switzerland; ^e^software μCT Tomography V5.4C, Scanco Medical AG, Brüttisellen, Switzerland; ^f^AMIRA 5.4.2, Visage Imaging GmbH, Berlin, Germany.

## Abbreviations

2D: Two-dimensional; 3D: Three-dimensional; HU: Hounsfield unit; ID: Identity; kVp: Kilovoltage peak; mA: Miliampere; mm: Milimetres; ms: Miliseconds; n: Number; rbd: Root bucco-distal; rbm: Root bucco-mesial; rd: Root distal; rm: Root mesial; rp: Root palatal; μCT: Micro-computed tomography; μm: Micrometres.

## Competing interests

Susan Kopke: No financial and non-financial competing interests exist.

Nina Angrisani: No financial and non-financial competing interests exist.

Carsten Staszyk: No financial and non-financial competing interests exist.

## Authors' contributions

SK designed the study, collected and processed the specimens, conducted the micro-computed tomography scans, assembled and analysed the data, drafted and wrote the manuscript. NA contributed to the study design and the micro-computed tomography examinations. CS contributed to the study design, helped to collect and process the specimens, contributed to data analysis and interpretation, and edited and revised the manuscript. All authors read and approved the manuscript.
